# The randomization-induced risk of a trial failing to attain its target power: assessment and mitigation

**DOI:** 10.1186/s13063-019-3471-8

**Published:** 2019-06-17

**Authors:** Hubert Wong, Yongdong Ouyang, Mohammad Ehsanul Karim

**Affiliations:** School of Population & Public Health, University of British Columbia, 2206 East Mall, Vancouver, BC V6T 1Z3 Canada

**Keywords:** Power, Attained power, Power distribution, Stepped-wedge design, Restricted randomization

## Abstract

Health researchers are familiar with the concept of trial power, a number that *prior to the start of a trial* is intended to describe the probability that the results of the trial will correctly conclude that the intervention has an effect. Trial power, as calculated using standard software, is an *expected power* that arises from averaging hypothetical trial results *over all possible treatment allocations that could be generated by the randomization algorithm*. However, in the trial that ultimately is conducted, only *one treatment allocation will occur*, and the corresponding *attained power* (conditional on the allocation that occurred) is not guaranteed to be equal to the expected power and may be substantially lower. We provide examples illustrating this issue, discuss some circumstances when this issue is a concern, define and advocate the examination of the *pre-randomization power distribution* for evaluating the risk of obtaining unacceptably low attained power, and suggest the use of randomization restrictions to reduce this risk. In trials that randomize only a modest number of units, we recommend that trial designers evaluate the risk of getting low attained power and, if warranted, modify the randomization algorithm to reduce this risk.

## Introduction

Clinical trialists are familiar with the concept of the power of a clinical trial. It is a number that *prior to the start of a trial* is intended to describe the probability that the results of a trial will correctly conclude that the intervention has an effect (i.e., will reject the null hypothesis of no effect when the null hypothesis is false). Conventionally, trials are designed to have 80% or 90% power. Given the high costs of conducting clinical trials, trial designers have a responsibility to ensure that the trial, in fact, attains the desired power. Imagine now that the power of your proposed trial is established, based on commonly used power calculation software, to be 80%. For concreteness, suppose it is a cluster-randomized controlled trial (cRCT) in which the cluster sizes vary substantially. At trial kickoff, the trial methodologist performs the randomization of the clusters. However, when the methodologist attempts to verify the power of the trial *based on the allocation that was obtained*, the power of the trial is determined to be only 75%. How could this happen and how might it have been avoided?

The fundamental issue is that trial power, as calculated using standard software, is an *expected power* that averages hypothetical trial results *over all possible treatment allocations that could be generated by the randomization algorithm*. However, in the trial that ultimately is conducted, only *one treatment allocation will occur*, and the corresponding *attained power* (conditional on the allocation that occurred) is not guaranteed to be equal to the target expected power and may be substantially lower. Hence, a trial may fail to attain adequate power because of bad luck. This problem arises because standard software packages typically require trial designers to make simplifying assumptions and to supply inputs that may not fully reflect the underlying characteristics and processes of the actual trial. Simple examples include (1) assuming that the sample size will be exactly equal in the two arms when the randomization algorithm has not been designed to guarantee this result and (2) using an average cluster size in the calculations for a cRCT even though the cluster sizes vary widely. Even when trial designers acknowledge and account for these types of issues through trial-specific power investigations (often via simulation), the calculations almost always continue to focus on determining the expected power and do not consider the variation in the attained power associated with different allocations. To our knowledge, no one has proposed a general approach for assessing the quantitative impact of varying allocation on attained power. As a result, the risk that a trial does not achieve the target power typically is not assessed. Moreover, if trial designers were able to identify the allocations that yield low attained power, they could modify the randomization algorithm to avoid those allocations and ensure that the trial achieves an acceptable attained power.

Through a series of examples, we illustrate why attained power may be substantially lower than the target expected power. We discuss general conditions when this issue is (or is not) a concern and advocate for examination of the distribution of the power prior to randomization as a general approach for evaluating the risk of obtaining unacceptably low attained power. Finally, we recommend the use of randomization restrictions to reduce this risk.

## Why and when you don’t get the power you think you’re getting

### Example 1: Parallel-arm trial with individual randomization

Consider a simple trial in which 40 participants are randomly assigned individually to one of two arms and the outcome is a continuous variable (e.g., systolic blood pressure). The data will be analyzed by using a two-sided, two-sample *t* test at the 5% significance level. Standard power calculation software shows that if the standardized effect size is 0.91 and 20 patients are allocated to each arm, then the expected power of this trial is 80%.

However, suppose that when the trial was conducted, the allocation algorithm did not ensure exact balance in the number randomized to each arm. For example, independent “coin flips” were used to allocate the participants and ultimately this process led to an unbalanced allocation with 15 and 25 participants in the two arms. An imbalance of this degree or greater is not a rare occurrence; the chance is 15.4%. If the values of the other parameters remain the same, it is straightforward to verify that with sample sizes of 15 and 25, the attained power is only 77.4%. If the imbalance were more extreme with sample sizes of 12 and 28 in the two arms (the chance of this degree of imbalance or greater is 1.7%), the attained power drops to 72.8%.

**Fig. 1:**
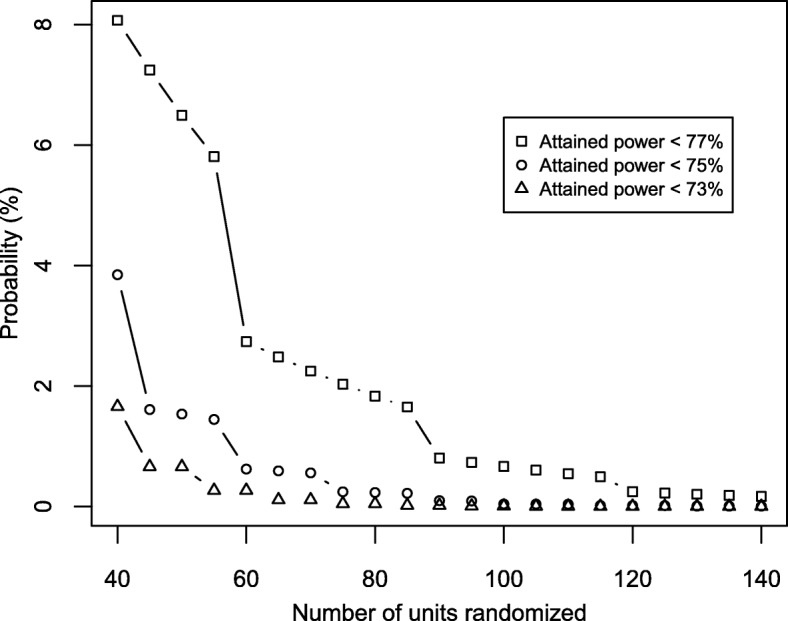
Risk of the attained power falling below threshold values (77%, 75%, or 73%) due to sample size imbalance as the number of units randomized (total sample size) increases in a parallel, two-arm, individually randomized trial using unrestricted randomization. Results were obtained under the condition that the power is 80% when the sample sizes are equal in the two arms. The risk of low attained power can be substantial with small sample sizes but decreases rapidly as the sample size increases. (The lack of smoothness is a consequence of discretization on the probabilities of obtaining allocations with different sample sizes.)

In this simple case, the solution to avoiding loss of power is evident. We need only ensure that the randomization algorithm allocates (nearly) equal numbers of participants to each arm. In practice, this balance typically is achieved by using block randomization. Note that the chance of getting an imbalance large enough to effect a given reduction in the attained power decreases rapidly as the sample size increases (Fig. 1). Hence, depending on what is judged an important reduction in attained power, block randomization may not be needed when the sample size is large. For example, the risk of the attained power falling below 75% is 3.8% when the sample size is 40 but is only 0.04% when the sample size is 100. However, with a sample size of 100, there remains a 0.7% risk that the attained power will fall below 77%.

### Example 2: Parallel-arm, cluster-randomized controlled trial (cRCT) with unequal cluster sizes

In a cRCT, groups (clusters) of participants are randomly allocated to treatments; as a result, everyone within a given cluster receives the same treatment. The literature on cRCTs is too extensive to cite here, but an introduction to the cRCT design can be found in Donner and Klar [1]. In a cRCT, it is common for the cluster sizes to be unequal. Methods and statistical software (available both online and in stand-alone packages) for sample size/power calculations for a cRCT are widely available for the case of *equal* cluster sizes and are less common for the case of unequal cluster sizes (see Rutterford et al. [2] for a review). Many of the approaches for unequal cluster sizes involve replacing the cluster sample size in the formulae for equal cluster sizes with an appropriate “effective cluster sample size” derived from a measure of the variability (e.g., the coefficient of variation) in the cluster sample sizes. However, to our knowledge, all of these methods and software provide calculations for only the *expected power*; the issue of varying *attained power* with varying allocation has not been fully addressed.

Suppose we are conducting a cRCT in 20 clusters. We expect that in each of six large, six medium, and eight small hospitals, the enrollments will be 160, 40, and 10 patients, respectively (yielding a coefficient of variation of 0.95). Under a specified set of parameters, including an intraclass correlation coefficient (ICC) of 0.005, an allocation that had perfect balance in the distribution of hospital sizes across the two treatment arms (i.e., three large, three medium, and four small hospitals in each arm) attains 80% power. However, if five large hospitals were allocated to the same arm (a 21.9% chance of occurring), the power would fall to 74%. If all six large hospitals were allocated to the same arm (a 3.1% chance of occurring), the power would fall to 68%.

Let us explain these dramatic drops heuristically. When the ICC is low, individual observations contribute nearly equally to the analysis. Hence, a large imbalance in the number of participants across the arms impacts the analysis much like large imbalances in individually randomized trials. Thus, instead of a comparison of 640 participants in each arm in the balanced case, the comparison becomes one between roughly 1000 versus 280 participants in the case with all six large hospitals in one arm. With a larger ICC, the drop in power is reduced and may even be negligible. With an ICC of 0.04, the powers are 79% or 78% when, respectively, five or six large hospitals are in one arm. This result is due to the “diminishing returns” in information that additional observations provide when the primary source of uncertainty is the variability across clusters rather than across participants within a cluster [3].

In this example, the appropriate way to restrict the randomization to avoid low attained power seems clear: ensure that the *distribution of cluster sizes* (not just the mean size) is similar in the two arms. The impact of doing so can be quantified by using the framework in the following example where varying cluster size could be viewed as an example of a cluster-level covariate which may be imbalanced across the two arms.

### Example 3: Parallel-arm trial with covariate adjustment

Assessments of attained power should consider balance not only in sample size but also in potentially important covariates. As in Example 1, consider a trial with 40 participants, individually randomly assigned to one of two arms, and a continuous outcome. We now assume that the randomization algorithm ensures equal sample sizes in the two arms, so given the same parameter values as before, the power of the trial should be 80%. However, suppose that there exists a binary baseline covariate which may be a determinant of the outcome but this covariate was not considered in the randomization algorithm. If this covariate turns out to be imbalanced across the treatment arms, we are obligated to adjust for it in the analysis in order to avoid potential confounding of the treatment effect estimate. Suppose that the covariate does not affect the outcome. If seven patients in one arm and 13 patients in the other arm have this attribute, the increase in the standard error of the treatment effect estimate due to the correlation between treatment arm and this covariate leads to a drop in the attained power to 77%. This reduction in the attained power is not a rare occurrence; there is a 6.9% chance of getting an imbalance this large (or greater). If six patients in one arm and 14 patients in the arm have this attribute (0.7% chance of occurrence), the attained power drops to 74%.

Methods for preventing covariate imbalance include stratified randomization and minimization [4, 5]. Whereas the discussion of the benefits of these methods often focuses on the goal of pre-empting potential confounding bias in treatment effect estimates, the impact on trial power has also been considered. See, for example, the literature cited within the reviews by Kahan and Morris [6] and Scott et al. [7].

As in Example 1, covariate imbalance typically is not an issue in individually randomized studies when a large number of units are randomized. The risk of an imbalance large enough to lead to a meaningful loss in the attained power decreases quickly as the number of units randomized increases.

Cluster-level covariate imbalance can also arise in cluster-randomized trials and is more common as the number of clusters randomized typically is modest. Previous works [8, 9] have shown that adjustment for a non-prognostic cluster-level covariate reduces the (expected) power. This loss in power, however, is greatly reduced if the randomization is constrained by dis-allowing allocations with large covariate imbalance [10]. The natural heuristic explanation is that the removed allocations predominantly are the ones with low attained power (analogous to the example above for the individually randomized trial), so their removal increases both the attained and the expected power. Software implementing covariate-balancing randomization algorithms is available [11, 12]. However, the next example involves a type of cluster-randomized trial design in which a cluster-level covariate *cannot be fully balanced* and for which covariate adjustment is a critically important determinant of the attained power when the number of clusters is small.

### Example 4: Stepped-wedge trial with unequal cluster sizes

A stepped-wedge trial is a special type of cluster-randomized trial. In the standard form of this design, every cluster begins with enrollment of participants into the control intervention and ends with enrollment into the experimental intervention. The object of randomization is the time point at which a cluster transitions from the control to the experimental intervention. The stepped-wedge trial design [13, 14] has received considerable attention in recent years. Methods and software for calculating power for various types (cross-sectional and cohort) of stepped-wedge trials with equal cluster sizes are becoming widely available [15, 16]. Kristunas et al. [17] reported that imbalances in cluster size do not result in a meaningful loss of power in cross-sectional stepped-wedge trials. More recently, Girling [18] derived analytic expressions for the efficiency of stepped-wedge and related designs with unequal cluster sizes relative to designs with equal cluster sizes while assuming that randomization was size-stratified so that the distribution of cluster sizes is the same in each treatment sequence (study arm). However, both of these studies focus solely on expected power. As in the preceding examples, we demonstrate that the attained power may vary substantially across allocations. One difference from the preceding examples is that it is not immediately obvious which allocations lead to low power.

For this example, we will adopt the expected cluster sample sizes from a real cross-sectional stepped-wedge trial under development. In this trial, there are 20 clusters (hospitals). If one assumes that enrollments will be approximately proportional to the population catchment areas of the hospitals, the expected cluster sample sizes range from six up to 86 for the 16 smallest clusters, and the sizes for the four largest clusters are 234, 215, 112, and 109. The coefficient of variation for these cluster sizes is 1.17. At each of five time points, four hospitals will transition from the control to the experimental intervention. For illustrative purposes, the results presented were obtained using an ICC of 0.05.

We can argue the dependence of the attained power on the allocations in at least two ways. The first argument is that the attained power will be higher when the large hospitals transition at the third (i.e., middle) step since these hospitals contribute the most information and their information is maximized by balancing the number of enrollments across the two interventions within these hospitals. However, it is vital to recognize that, in a stepped-wedge trial, participant treatment allocation is inherently highly correlated with time *by design*. Participants enrolled during the early periods predominantly will receive the control intervention while participants enrolled during the later periods predominantly will receive the experimental intervention. For a five-step balanced (equal cluster size) design, the Pearson correlation coefficient is 0.68 [19]. Thus, if there is any possibility that a secular time trend exists in the outcomes, we are obligated to adjust for the time (step) in the analysis in order to remove potential bias. As discussed in Example 3, adjustment for a covariate correlated with treatment allocation can reduce the attained power and the magnitude of the reduction increases with higher correlation. Hence, a second argument is that we can reduce the loss in attained power by reducing the correlation between participant treatment allocation and time. This correlation is lower when the large hospitals transition at the first or last steps. In this example, the correlation coefficient will be roughly 0.57 when the four largest hospitals transition at the first or last steps but will be roughly 0.78 when these hospitals transition at the middle step. Through simulation, we observed that whereas this trial would achieve 80% power if the randomization algorithm restricted the four largest hospitals to transition at the first or last steps (two hospitals at each of these steps), it would achieve only 73% power if the algorithm restricted those hospitals to transition at the middle step. Thus, it appears that, at least in this example, reducing the correlation between treatment allocation and time is more important than balancing observations in the large clusters. Other authors have reported related results. Lawrie et al. [20] showed that in a cross-sectional setting with *equal* cluster sizes, the most efficient design allocates more clusters to transition at the first and last steps than in the intermediate steps. Li et al. [21] extended this result to the cohort setting. Girling and Hemming [22] showed that if (equal sized) clusters are allowed to enroll purely into either the control condition or the intervention condition, as is done in a parallel cRCT, the most efficient design is obtained by allocating roughly two thirds of the clusters using the stepped-wedge layout and one third using the parallel layout. Though not discussed in these papers, it can be shown that in each case the treatment-time correlation decreases when moving from the standard stepped-wedge design to the most efficient design. Thus, it appears that this correlation parameter is a crucial determinant of power (attained and expected) across a wide range of settings, although its absolute impact and relative importance compared with other factors affecting power have yet to be investigated.

## The pre-randomization power distribution and randomization restriction

**Fig. 2:**
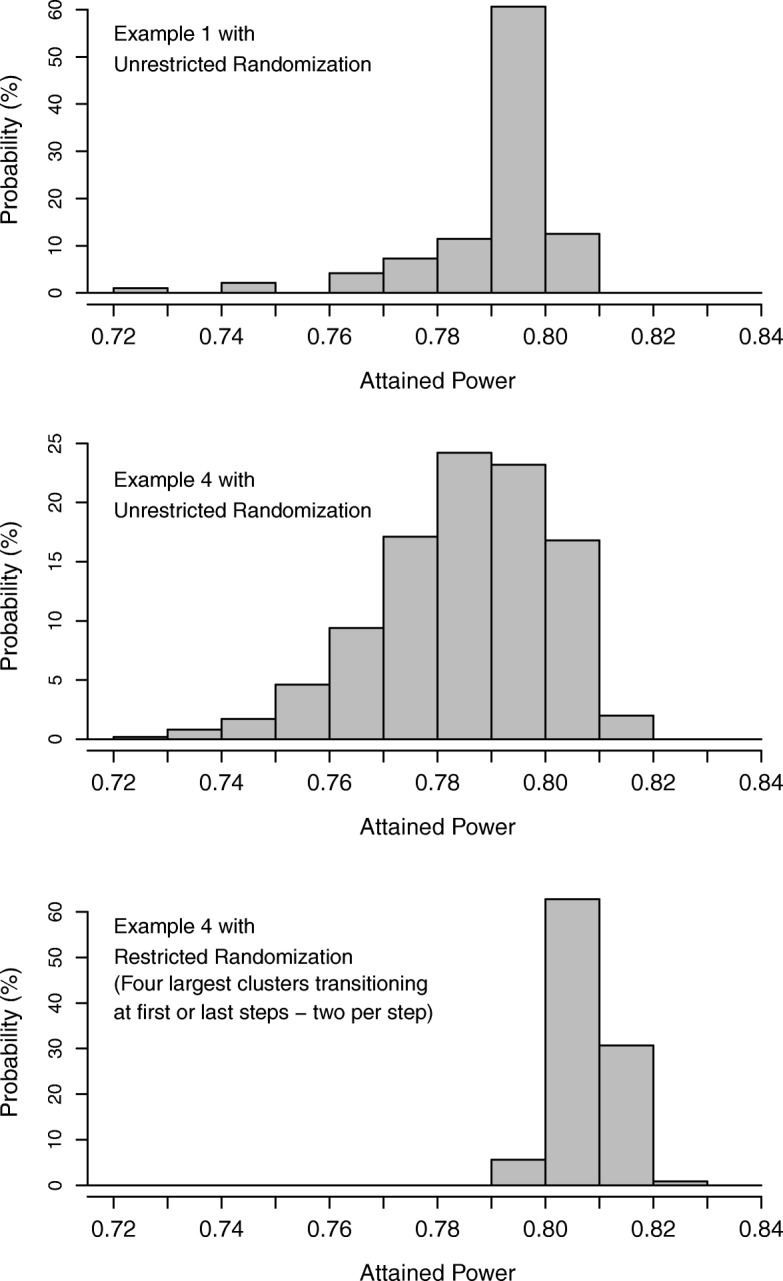
Density plot (histogram) of the power distribution. Upper panel: Individually randomized trial from Example 1 with unrestricted randomization. Middle panel: Stepped-wedge trial from Example 4 with unrestricted randomization. Lower panel: Stepped-wedge trial from Example 4 with restricted randomization (four largest clusters transitioning to intervention at the first or last step, two clusters at each of these steps).

In complex trial designs, many factors can interact to affect the attained power, and it may not be easy to determine the risk that a specified randomization algorithm will lead to low attained power. One approach to determining this risk is to construct the algorithm’s *pre-randomization power distribution* (*power distribution* [*PD*] hereafter). Conceptually, this distribution can be obtained by calculating the attained power associated with every possible allocation that can be generated by the randomization algorithm and constructing a probability distribution (either the density or the cumulative distribution, as needed) from this collection of attained powers. By examining the PD, one can determine, for any given threshold, the probability that the attained power will fall below that threshold. For example, the second and third columns in Table 1 display the probability that the attained power will fall below selected thresholds based on the PD for the unrestricted randomization algorithms used in Examples 1 and 4. The PD can also be summarized graphically (Fig. 2, upper and middle panels). If the risk based on the PD is deemed unacceptable, the randomization algorithm could be modified. As noted earlier for Example 1, merely restricting the randomization to ensure equal sample sizes would guarantee 80% attained power. For Example 4, the risk of an attained power below 75% is 2.7% (column 3, Table 1) when unrestricted randomization is used. But if allocations are restricted to include only the ones in which the four largest clusters transition at the first or last steps (two clusters at each of these steps), the risk drops to zero and in fact this restriction guarantees a power of at least 79% (column 4, Table 1 and lower panel, Fig. 2).

**Table 1:** The risk that the attained power will fall below selected threshold values

Threshold	Risk that the attained power falls below the threshold value		
	Example 1 (unrestricted)^a^	Example 4 (unrestricted)^b^	Example 4 (restricted)^c^
72%	0.6%	0.0%	0.0%
73%	1.7%	0.2%	0.0%
74%	1.7%	1.0%	0.0%
75%	3.8%	2.7%	0.0%
76%	3.8%	7.3%	0.0%
77%	8.1%	16.6%	0.0%
78%	15.4%	33.5%	0.0%
79%	26.8%	57.9%	0.0%
80%	87.5%	81.0%	5.4%
81%	100.0%	97.8%	68.0%
82%	100.0%	100.0%	99.0%
83%	100.0%	100.0%	100.0%

^a^Results are for unrestricted randomization in Example 1.

^b^Results are for unrestricted randomization in Example 4.

^c^Results are for a randomization algorithm that allows only allocations with the four largest clusters transitioning at the first or last steps (two clusters at each of these steps) in Example 4.

We do not make recommendations about what are appropriate minimum threshold power values or acceptable risks; these are subjective judgments and their importance will be context-dependent.

## Discussion

The examples are presented as “proof of principle” that the attained power of a trial may be substantially below the target power that was generated by using standard power calculations. The magnitude of this risk is a complex function of the number of units randomized, the type of study design (individually randomized, cRCT, stepped-wedge, etc.), the specifications of the study design (e.g., randomization restrictions and number of steps in a stepped-wedge), and population characteristics (e.g., ICC and variation in cluster sizes). We have shown that when the number of units randomized is large in an individually randomized trial, the risk of this event is sufficiently low that it might be ignorable. Similar results should hold for cluster-randomized trials, although work is needed to quantify the impact of cluster-design parameters on the conclusions. However, the conditions under which this risk should not be ignored do arise in real trials; the calculations presented in Example 4 were a key determinant in our decision to adopt the restricted randomization scheme for that trial. Whenever the number of units randomized is modest, trial designers should investigate the risk that the randomization algorithm will yield an unacceptably low attained power. We have proposed evaluating that risk using the power distribution. If that risk is unacceptably high, then the problem allocations should be identified, and the randomization algorithm should be modified to exclude them. Implementing restrictions to allocations with high attained power will also increase the expected power. In Example 4, using the restricted randomization algorithm raised the expected power to 80.8% from 78.5% for the unrestricted algorithm. If a substantial gain in expected power can be achieved, it potentially allows for increased efficiency via a reduction in overall sample size.

One concern regarding restricting randomizations is that the randomization subspace may include pairs of clusters that tend to be in the same arm (treatment sequence). Conceptually, this could lead to violation of assumptions needed for valid randomization inference [23]. Greene [11] discusses an example where this may be a concern. The conditions under which this problem occurs are not apparent and warrant further exploration.

Analytic formulae for calculating the PD are not easily obtained except for the simplest trial designs/randomization algorithms, so at least some of the calculations will involve simulation. A simulation approach also has the advantage that it can accommodate arbitrarily complex trial designs and analyses with relatively modest programming effort. These simulations can involve substantial computational time since a separate attained power needs to be calculated for each possible allocation. A “brute force” approach (i.e., one without much effort put into reducing the computation time) used to obtain each of the PDs in Example 4 required about 90 h on a desktop PC to evaluate the attained power for 1000 randomly sampled allocations with 10,000 simulation runs per allocation.

Our primary aim here has been to raise conceptual awareness of an important design consideration that seems to have been overlooked, rather than to provide solutions for specific trial designs. Much work needs to be carried out to support trial designers in selecting optimal restricted randomization algorithms. Evaluation of the attained power for every allocation may not be feasible when the number of possible allocations is large (e.g., with 20 unique cluster sizes in Example 4, the number of unrestricted allocations is greater than 300 billion). Strategies, efficient algorithms, and software to enable accessible and timely calculation of PDs need to be developed. In addition, trial designers need guiding principles that enable them to quickly target the most promising randomization restrictions for different types of designs. One natural way to facilitate the use of the PD would be to embed methods for determining PDs into the framework of Li et al. [10] with options for choosing a PD-based metric (e.g., exclude allocations with attained power below a threshold value) that could be used, alongside covariate balance or other metrics, to restrict randomization and assess the acceptability of a proposed randomization algorithm. Availability of these tools and guidelines will enable researchers to ensure that the power of a trial is not compromised by chance.

## Data Availability

Not applicable. Simulation codes are available from the authors on request.

## Abbreviations

cRCT: Cluster-randomized controlled trial
ICC: Intraclass correlation coefficient
PD: (Pre-randomization) power distribution
